# Engineered cyclodextrin glucanotransferases from *Bacillus* sp. G‐825‐6 produce large‐ring cyclodextrins with high specificity

**DOI:** 10.1002/mbo3.757

**Published:** 2018-10-25

**Authors:** Christian Sonnendecker, Susanne Melzer, Wolfgang Zimmermann

**Affiliations:** ^1^ Department of Microbiology and Bioprocess Technology, Institute of Biochemistry Leipzig University Leipzig Germany; ^2^ Present address: Clinical Trial Centre Leipzig, Medical Faculty Leipzig University Leipzig Germany

**Keywords:** *Bacillus*, cyclodextrin glucanotransferase, cyclodextrins, protein engineering, side‐directed mutagenesis

## Abstract

Cyclodextrin glucanotransferases (CGTases) synthesize cyclic oligosaccharides (cyclodextrins, CD) from starch. A CGTase from *Bacillus *sp. G‐825‐6 was engineered by site‐directed mutagenesis at two positions by the construction of the variants Y183W, Y183R, D358R, Y183W/D358R, and Y183R/D358R. Among CD composed of 7–12 glucose units (CD7–CD12), Y183W mainly produced CD8. Y183R had completely lost its ability to synthesize CD7, and CD8 and the larger CD were the only cyclic oligosaccharides produced. D358R also formed mainly CD8–CD12 during a reaction time of 24 hr. The double mutant Y183W/D358R showed combined characteristics of the single mutations with very low CD7 cyclization activity and an increased formation of the larger CD. The results show that CGTases synthesizing mainly CD8–CD12 can be constructed allowing a convenient production of larger CD in significant amounts as host molecules in supramolecular complexing reactions.

## 1 INTRODUCTION

Cyclodextrin glucanotransferases (CGTases) are members of the GH13 α‐amylase family and synthesize cyclodextrins (CD) from starch substrates. They catalyze intra‐ and intermolecular transglycosylation reactions with an α‐retaining double displacement mechanism performed by a catalytic triad composed of three conserved carboxylates (van der Veen, Alebeek, Uitdehaag, Dijkstra, & Dijkhuizen, 2000). CGTases have been detected in bacteria such as *Bacillus*, *Klebsiella*, *Thermoanaerobacter* and also in archaea such as *Thermococcus *species (Leemhuis, Kelly, & Dijkhuizen, 2010). These CGTases showed differences in their pH‐ and temperature optima, substrate and product specificity, and in their catalytic efficiency. CGTase is a five domain protein where domains A1 and A2 form a catalytically active (β/α)8‐barrel. Besides the cyclization reaction, where CD are formed by an intramolecular transglycosylation of a linear α‐1,4 glucan chain, three further reactions are catalyzed by CGTases. In a coupling reaction, cyclic glucan substrates are cleaved and transferred to a linear glucan acceptor. Linear glucans can also be transferred to a linear acceptor by a disproportionation reaction. Furthermore, in a hydrolysis reaction, a linear glucan cleavage product is transferred to water as the acceptor by the CGTase (Qi & Zimmermann, 2005). Glucan binding subsites play an important role in the glycosylation reactions of CGTases influencing the size of the CD products (Davies, Wilson, & Henrissat, 1997; Strokopytov et al., 1996; Uitdehaag, Alebeek, Veen, Dijkhuizen, & Dijkstra, 2000; Uitdehaag, Kalk, Veen, Dijkhuizen, & Dijkstra, 1999).

Since CD form specific inclusion complexes with organic and inorganic guest molecules, they have found a broad range of applications in the pharmaceutical industry to increase the solubility, stability, and bioavailability of drugs (Del Valle, 2004; Loftsson & Brewster, 1996). CD are also used for the protection of food components against oxidation and light‐induced reactions or for the elimination of undesired tastes and smells (Szente & Szejtli, 2004). Compared to CD composed of 6 and 7 glucose units (CD6 and CD7) formed by most CGTases as their main products, CD8 features a larger hydrophobic cavity allowing the formation of complexes with more space‐demanding guest molecules (Endo, Zheng, & Zimmermann, 2002; Li et al., 2007). CGTases also produce large‐ring CD (LR‐CD) with a degree of polymerization (DP) of more than 60 (Terada, Yanase, Takata, Takaha, & Okada, 1997). LR‐CD with a DP of 17 and higher can also be formed by bacterial amylomaltases (4‐α‐glucanotransferases) or by the plant‐derived D‐enzyme (Roth et al., 2017; Terada, Fujii, Takaha, & Okada, 1999). While CD9 is a doughnut‐shaped molecule like CD6, CD7, and CD8, CD14 shows a boat‐like structure and CD24 contains helix‐like backbone elements (Gotsev & Ivanov, 2007). LR‐CD are produced during the initial cyclization reaction and are subsequently converted by the CGTase to form linear products and smaller CD (Terada et al., 2001). As a result, only small amounts of LR‐CD can usually be obtained by a CGTase reaction with starch. In addition, the isolation of single LR‐CD from the reaction mixtures in a downstream process is time‐consuming and costly, and only milligram quantities have been prepared previously using chromatographic procedures (Endo et al., 1998; Biwer, Antranikian, & Heinzle, 2002; Endo, Ueda, Kobayashi, & Nagai, 1995). Since several LR‐CD have shown interesting complex‐forming abilities (Assaf, Gabel, Zimmermann, & Nau, 2016; Endo & Ueda, 2004; Endo et al., 2002), it is of high interest to identify CGTases with high product size specificity yielding predominantly larger CD of a defined size (Chen et al., 2015; Kaulpiboon, Pongsawasdi, & Zimmermann, 2010; Li et al., 2014).

The objective of this study was to increase the product specificity of a CGTase from *Bacillus* sp. G‐825‐6 by site‐directed mutagenesis to obtain enzyme variants specifically synthesizing larger CD.

## 2 EXPERIMENTAL PROCEDURES

### 2.1 DNA manipulation and heterologeous protein expression

A pET20b(+) expression vector encoding the DacD signal peptide fused to the mature CGTase G825‐6 sequence from *Bacillus* sp. G825‐6 (GenBank: AB201304) was used as template (Sonnendecker et al., 2017). Mutagenic primers were designed and mutagenesis was performed as described elsewhere (Liu & Naismith, 2008). Plasmid isolation and DNA sequencing of the CGTase encoding gene were performed to confirm the correct incorporation of mutations. Recombinant protein expression in *Escherichia coli* BL21(DE3) and purification based on starch adsorption were performed as described elsewhere (Sonnendecker et al., 2017).

### 2.2 Protein quantification

The Bradford assay was used for protein quantification with RotiQuant and BSA as standard (Carl Roth, Karlsruhe, Germany) in 96‐well microplate format and samples were measured at 590 and 450 nm (Zor & Selinger, 1996). Purified protein samples were analyzed by gel electrophoresis using 12% polyacrylamide gels and 4% stacking gels by adding 1 µg of purified protein per lane. Pierce Unstained Protein MW Marker was used for size determination (Thermo Scientific, Schwerte, Germany).

### 2.3 CD synthesis and LR‐CD isolation

Soluble starch solution (20 g/L) in CGTase buffer (25 mM Tris‐HCl, 10 mM KCl, 5 mM MgCl_2_, pH 8.5) was boiled to solubilize the starch (soluble potato starch, CAS9005‐84‐9; Merck KGaA, Darmstadt, Germany). One millilitre of substrate solution was incubated at 50°C and 0.2 µg purified CGTase was added to start the cyclization reaction. To study the effect of the enzyme concentration on CD product formation, 0.1, 0.2, 0.4, 0.8, and 1.6 µg purified CGTase were added to 1 ml of 20 g/L starch substrate in CGTase buffer containing 5 vol% ethanol and incubated for 8 hr. Reactions were stopped by adding 100 µl sample to 100 µl 0.2 M acetic acid buffer, pH 4.5 followed by 10 min at 95°C to inactivate the enzyme. 0.25 U Glucoamylase (Sorachim, Lausanne, Switzerland) was added and the mixture was incubated at 40°C over night to degrade the linear glucans to glucose. The samples were heated to 95°C for 10 min to inactivate the enzyme and centrifuged at 18,000 *g* for 5 min prior to analysis. The determination of Michaelis–Menten enzyme kinetics was performed using soluble starch concentrations in the range of 1–20 g/L with a reaction time of 1 hr. Nonlinear regressions (*N* = 3 ± standard error) were performed using GraphPad Prism 7 (GraphPad Software Inc., La Jolla, CA, USA).

The synthesis of a LR‐CD mixture was performed with the CGTase variant Y183R (60 µg) and 1 L of starch substrate as described above. The residual starch was removed by centrifugation, followed by a glucoamylase treatment and a fivefold concentration. The CD were then precipitated by adding five volumes of acetone to the supernatant. After standing for 18 hr at room temperature, the product was washed twice with acetone and freeze dried. The resulting CD mixture was dissolved in CGTase buffer to a final concentration of 10 g/L and used as substrate for coupling reactions with the CGTase 825‐6 and the variant Y183R. The reactions were performed as described above for the cyclization reactions.

### 2.4 Analysis of CD by HPAEC

The mixtures of synthesized CD6–CD12 were analyzed by high performance anion exchange chromatography (HPAEC) with pulsed amperometric detection as previously described (Melzer, Sonnendecker, Föllner, & Zimmermann, 2015). Injection was performed with a 25 µl sample loop. Purified CD6, CD7, and CD8 (Wacker Chemie AG, Munich, Germany) were used for calibration in a range of 5–100 µg/ml CD. LR‐CD concentrations were calculated using CD8 calibration curves.

### 2.5 Protein structure modeling

The template pdb (protein data bank) structure 1cxk from *Bacillus circulans* 251 CGTase was used to obtain ligand coordinates for a glucan substrate with a DP of 9 (Uitdehaag, Kalk, et al., 1999). A CGTase G825‐6 model was generated with SWISS‐MODEL (Bordoli et al., 2009) by using the pdb structure 4jcm from the *Bacillus clarkii* CGTase as the template. Modeling was performed with PyMol molecular graphic system (v0.99; Schrödinger, LCC). The numbering of the amino acid residues of the variants refers to the sequence of the mature CGTase G825‐6 sequence, where position 183 corresponds to position 195 in the *B. circulans *CGTase (Hirano et al., 2006).

## 3 RESULTS

### 3.1 Construction and purification of CGTase variants

Single mutations were introduced by site‐directed mutagenesis to generate the CGTase variants Y183W, Y183R, and D358R (Parsiegla, Schmidt, & Schulz, 1998; Wind, Uitdehaag, Buitelaar, Dijkstra, & Dijkhuizen, 1998; Xie, Song, Yue, Chao, & Qian, 2014). Double mutants 183W/D358R and Y183R/D358R were constructed in a second step. The variants were expressed in *E. coli* and the extracellular protein fractions were purified to apparent homogeneity confirmed by SDS‐PAGE (Supporting Information Figure S1).

### 3.2 Cyclization activity of the CGTase constructs at initial reaction conditions

Equimolar concentrations of proteins of the CGTase G‐825‐6 and the variants were used for a comparison of their reaction kinetic parameters (Table 1). While in the wild‐type enzyme, the ratio of the turnover numbers between CD7:CD8 was about 1:3, this drastically changed to a ratio of 1:58 in the variant Y183W. The variant Y183R produced CD8–CD12 with similar turnover numbers while it completely lost the ability to form CD7. The variant D358R showed the highest turnover number for the synthesis of CD8. The *k*_cat_ for the formation of CD7 and LR‐CD was six times and two times lower than the *k*_cat_ for CD8, respectively.

**Table 1:** Michaelis–Menten kinetics of the CD7–CD12 synthesis reactions catalyzed by the CGTase G‐825‐6 and the constructed variants

	*K*_m_ (g/L)	*k*_cat_ (s^−^^1^)
CGTase G825‐6		
CD7	4.1 ± 0.66	21.9 ± 1.27
CD8	5.1 ± 0.84	65.0 ± 4.23
CD9	4.0 ± 0.75	11.3 ± 0.75
CD10	3.6 ± 0.67	7.9 ± 0.50
CD11	4.3 ± 0.80	7.8 ± 0.53
CD12	4.6 ± 0.83	7.1 ± 0.48
Y183W		
CD7	0.3 ± 0.09	0.9 ± 0.02
CD8	5.2 ± 0.66	49.5 ± 2.23
CD9	3.6 ± 0.49	10.1 ± 0.43
CD10	0.4 ± 0.37	3.5 ± 0.37
CD11	2.3 ± 0.31	4.4 ± 0.15
CD12	2.4 ± 0.32	3.9 ± 0.14
Y183R		
CD8	11.3 ± 1.86	12.3 ± 1.05
CD9	20.2 ± 4.15	11.1 ± 1.43
CD10	16.7 ± 3.03	9.5 ± 1.02
CD11	23.9 ± 4.94	11.9 ± 1.63
CD12	28.7 ± 6.05	12.3 ± 1.80
D358R		
CD7	3.6 ± 0.45	1.9 ± 0.07
CD8	9.2 ± 1.10	12.9 ± 0.70
CD9	8.5 ± 0.91	6.4 ± 0.30
CD10	6.6 ± 0.72	6.3 ± 0.27
CD11	9.8 ± 0.89	7.4 ± 0.32
CD12	9.3 ± 0.82	6.0 ± 0.24

Note

Soluble starch was used as substrate. Nonlinear regression was performed to determine *K*_m_ and *k*_cat_. *N* = 3, mean ± standard error.

### 3.3 The CD product size spectrum of the CGTase G‐825‐6 and the engineered variants

**Figure 1:**
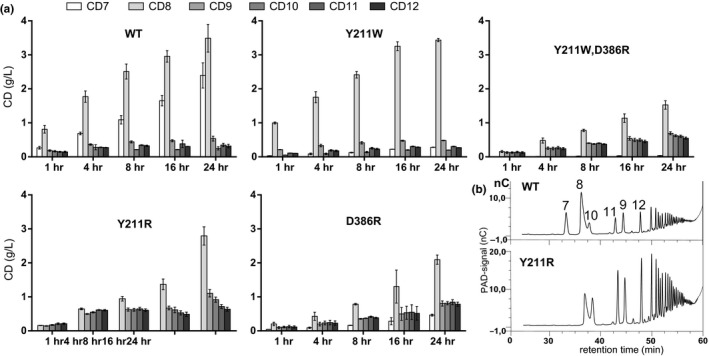
Cyclodextrins (CD) product size spectrum obtained with the CGTase G‐825‐6 and the constructed variants. (a) CD concentrations in g/L synthesized within a reaction time of 1, 4, 8, 16, and 24 hr are shown. *N* = 3, mean ± *SD*. (b) HPAEC chromatograms of CD produced by the CGTase G‐825‐6 (1:40 dilution) and by Y183R (1:20 dilution) within a reaction time of 8 hr. CD sizes are indicated by numbers

To evaluate the overall formation of CD by the CGTases over a reaction time of 24 hr, their product spectra obtained at different reaction times were analyzed (Figure 1). The CGTase G‐825‐6 synthesized mainly CD7 and CD8 during the 24 hr reaction together with small amounts of CD10 to CD12. CD6 was not detected as a product. A total of 36.7% of the starch substrate was converted to CD7–CD12 after 24 hr of reaction. The substitution of Tyr to Trp at the position 183 in the variant Y183W resulted in a drastic decrease in the amount of CD7 produced. After 24 hr of reaction, CD8 was detected as the main product. The synthesis of CD9–CD12 was not significantly affected by this amino acid substitution. The variant converted 25% of the starch substrate to CD within 24 hr of reaction. By replacing Tyr with Arg at the position 183, the variant Y183R produced mainly CD8 and higher amounts of CD9–CD12 compared to CGTase G‐825‐6 while CD7 was not detected as a product. This variant converted 31% of the starch substrate to CD within 24 hr of reaction. By substituting Asp by Arg at position 358, an increase of the amounts of LR‐CD synthesized was detected and the product spectrum was shifted toward the larger CD. This variant converted 29% of the starch substrate to CD within 24 hr of reaction. The variant Y183W/D358R combined the properties of Y183W and D358R in regard to a very small amount of CD7 formed and an increased synthesis of LR‐CD. However, its total cyclization activity was lower compared to Y183W and D358R. This variant converted 20% of the starch substrate to CD within 24 hr of reaction. The variant Y183R/D358R almost completely lost its cyclization activity. Only 1.3% of the starch substrate was converted to CD within a reaction time of 24 hr and its CD product size spectrum was similar to the variant Y183R.

### 3.4 Effect of the enzyme concentration on the CD product spectrum

**Figure 2:**
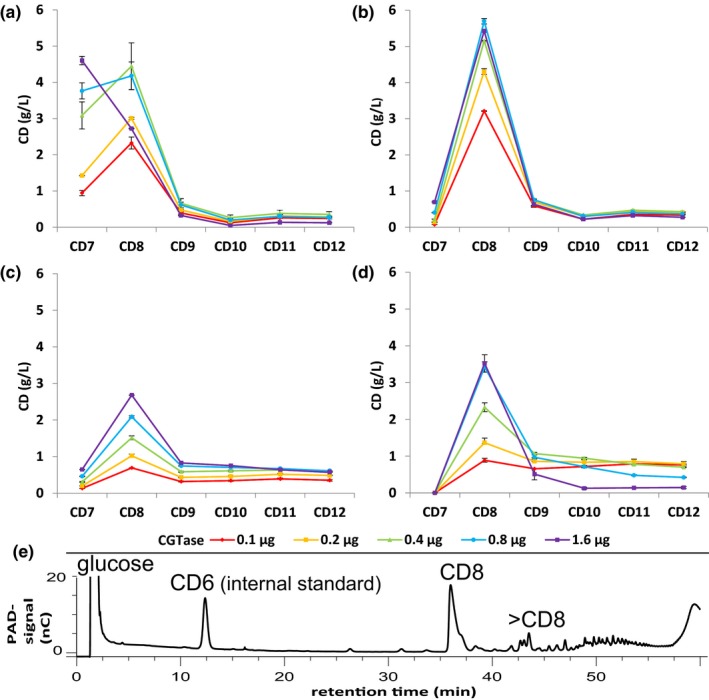
Effect of the enzyme concentration on the synthesis of cyclodextrins (CD) by CGTase G‐825‐6 and the variants. Purified CGTase (0.1–1.6 µg) was incubated for 8 hr with 20 g/L soluble starch; cyclic products were analyzed by HPAEC‐PAD. The amounts of CD7–CD12 produced are shown in g/L. (a) CGTase G‐825‐6, (b) Y183W, (c) D358R, (d) Y183R. *N* = 2, mean ± *SD*. (e) HPAEC‐PAD chromatogram of CD products formed by 1.6 µg/ml Y183R‐CGTase. After glucoamylase treatment, the sample was diluted 1:20. CD6 (50 mg/L) was used as internal standard

The effect of the enzyme concentration on the synthesis of CD of different sizes is shown in Figure 2. Within 8 hr of reaction, the CGTase G825‐6 accumulated CD8 as the main CD product. However, at higher enzyme concentrations, the amount of CD8 declined. CD7 became the main product and only small amounts of LR‐CD were produced. In contrast, the amounts of CD8 synthesized by the variants Y183R, Y183W, and D358R did not decline with higher enzyme concentrations and CD7 did not accumulate. Y183R formed almost equal amounts of LR‐CD and CD8 with a low enzyme concentration and almost only CD8 with an enzyme concentration of 1.6 µg/ml.

### 3.5 Changes of the size spectrum of a LR‐CD mixture by the CGTase G‐825‐6 and the variant Y183R

**Figure 3:**
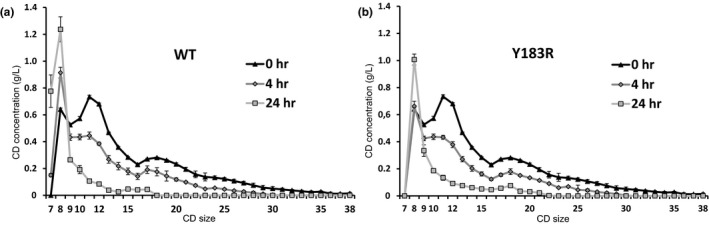
Conversion of a LR‐CD mixture (CD8–CD38) by the coupling reaction of the CGTase G‐825‐6 (a) and the variant Y183R (b). The coupling substrate (10 g/L) consisted of LR‐CD and glucose. CD products were analyzed by HPAEC after 0, 4, and 24 hr of reaction. *N* = 3, mean ± *SD*

When a CD mixture consisting of CD8–CD38 and glucose as acceptor was used as substrate for coupling reactions of the CGTase G‐825‐6 and the variant Y183R, it was observed that the largest CD were converted first (Figure 3). Y183R accumulated CD8 without the formation of CD7 resulting in a CD product mixture containing a high proportion of CD8, whereas CGTase G‐825‐6 converted the LR‐CD substrate to CD7.

## 4 DISCUSSION

### 4.1 CGTase G‐825‐6 as a template for the construction of variants producing LR‐CD

Previous studies of CGTases have shown that the amino acid substitutions Y195W (corresponding to 188W/R of the *Bacillus obhensis* CGTase and Y183 of the CGTase G825*‐*6) (Table 2) and D371R of the CGTase from *Thermoanaerobacterium thermosulfurigenes* (Wind et al., 1998), corresponding to D358 of the CGTase G825*‐*6, resulted in a shift of the produced CD spectrum toward larger CD. The CGTase from *B. clarkii *(Takada, Nakagawa, & Yamamoto, 2003) synthesizes larger amounts of CD8 but has the disadvantage to also produce CD6. Likewise, the CGTase from *B. obhensis* also forms larger amounts of CD7 besides CD8 (Sin, Nakamura, Kobayashi, Masaki, & Uozumi, 1991). The LR‐CD formed by these CGTases initially are rapidly degraded by their coupling activity. We selected the CGTase G‐825*‐*6 from a *Bacillus* species as the template for the construction of variants synthesizing larger CD since the enzyme is mainly forming CD8 without any CD6 (Hirano et al., 2006).

**Table 2:** Effects of the substitution of a Tyr residue located near the active site and a Asp residue at the glucan binding site −3 of CGTases

Reference	CGTase	Amino acid substitution	CD formation (%)^a^	CD products obtained		
				CD6	CD7	CD8
Sin et al. (1991)	*Bacillus obhensis*	Y188	100	0	30	5
		Y188W	77	0	15	22
		Y188R	49	0	7	10
Penninga et al. (1995)	*Bacillus circulans* no. 251	Y195	100	13	64	23
		Y195W	85	18	63	19
Parsiegla et al. (1998)	*B. circulans* no. 8	Y195	100	10	64	20
		Y195W	39	5	35	50
Xie et al. (2014)	*Paenibacillus *sp. 602‐1	Y195	100	83	10	7
		Y195W	110	62	21	17
		Y195R	36	35	15	50
Wind et al. (1998)	*Thermoanerobacterium thermosulfurigenes *EM1	D371	100	28	58	14
		D371R	83	6	68	26

a Percent conversion of substrate to CD. Wild‐type enzyme = 100%

### 4.2 CGTase variants show differences in substrate binding and turnover rates for CD formation

**Figure 4:**
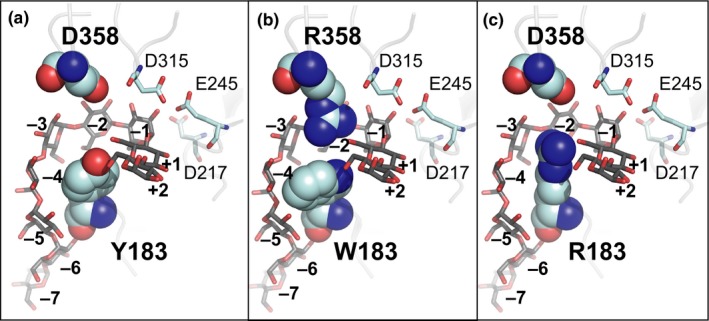
Models of the CGTase G‐825‐6 and the constructed variants showing the catalytic triad in relation to a maltononaose bound from binding site +2 to −7. Amino acid residues 183 and 358 are shown in spheres. The catalytic triad D217, E245, and D315 is shown as sticks. (a) CGTase G‐825‐6 with the amino acid residues Y183 and D358; (b) variant W183/D358R; (c) variant Y183R

**Figure 5:**
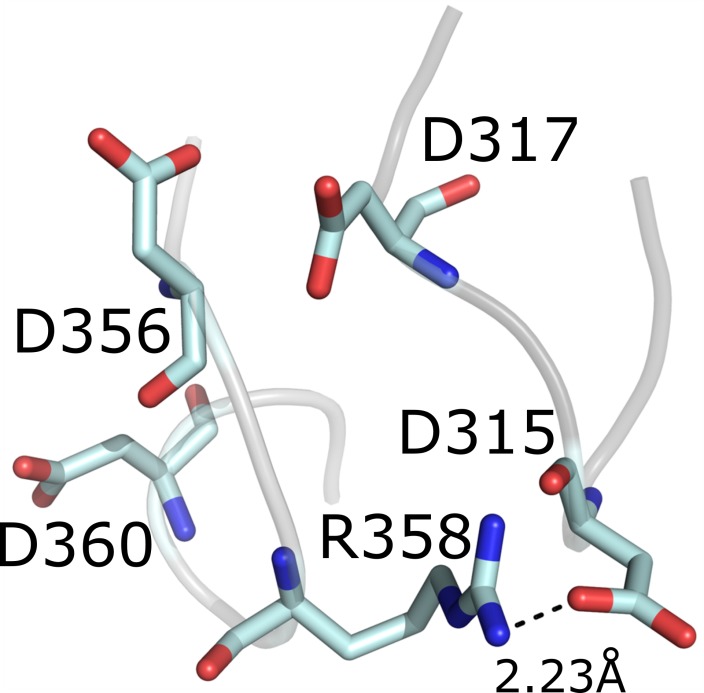
Possible salt bridge between the amino acid residues R358 and D315 of the CGTase. Distance measurement shown in dashed line

Mutations resulting in higher amounts of LR‐CD produced showed higher *K*_m_ values for their synthesis indicating a less efficient substrate binding due to the amino acid substitutions (Table 1). Y183W and D358R also showed low *K*_m_‐values for the formation of CD7 however with a much reduced *k*_cat_ value. It is possible that maltoheptaose could bind efficiently in the variants Y183W and Y183R but the winding process to form the cyclic product was disturbed. The correct positioning of the glucan intermediate could therefore be essential for the formation of a CD of a specific size and might explain the effect that CD7 could not be synthesized efficiently by these two variants. In the variant D358R, R358 could have a direct effect on the initial substrate binding by sterically blocking the binding at subsite −2/−3 explaining the increased *K*_m_ values of the variant D358R for the synthesis of CD8–CD12 (Figure 4). The more bulky residues R183 and R358 may force the substrate chain into conformations where more glucose residues can bind during the enzymatic cleavage explaining the observed increase in the sizes of the CD products formed. A corresponding mutation (D371R) of a CGTase from *T. thermosulfurigenes *EM1 also led to a weaker binding of maltohexaose and promoted the binding of maltononaose at the glucan binding subsite −2 (Wind et al., 1998). As a result, CD7 and CD8 were formed instead of CD6. We could confirm this effect, however by using the CGTase G825*‐*6 as template, we obtained much higher amounts of CD8 and LR‐CD. D358R may also influence D315, a catalytically active residue (Uitdehaag, Mosi, et al., 1999), by allowing the formation of a salt bridge and thereby reducing the turnover rate of the enzyme (Figure 5). A distance of 2.2 angstrom as indicated in the model would be sufficient for the formation of a salt bridge (Kumar & Nussinov, 2002).

### 4.3 Engineered CGTase G‐825‐6 variants display a unique CD product specificity

The number of glucose units covalently bound to the active site of the CGTase during the cleavage of the glucan chain has been suggested to determine the size of the CD formed (Uitdehaag, Kalk, et al. (1999), Uitdehaag, Mosi, et al. (1999), Uitdehaag et al. (2000). Mutations aiming to increase the size of the CD formed should therefore allow the initial binding of a higher number of glucose units of the substrate at the active site of the enzyme. As suggested previously, Tyr or Phe located near the active site of the CGTase play a crucial role in controlling the cyclization activity and CD product specificity of the enzyme (Nakamura, Haga, & Yamane, 1994; Parsiegla et al., 1998; Penninga et al., 1995; Sin et al., 1991; Xie et al., 2014). The effects of substitutions near the active site of various CGTases are summarized in Table 2. These substitutions affected the CD product specificity by favoring the formation of larger CD, however also resulting in a lower conversion rate of the starch substrate.

A high specificity for the synthesis of CD8 was achieved by replacing Tyr with the bulky aromatic Trp, while a higher specificity for CD9–CD12 was observed as the result of a replacement with the bulky, highly flexible, and positively charged Arg in the G825‐6 CGTase (Figure 4). Penninga et al. (1995) have likewise described the formation of CD9–CD12 following the replacement of Y195 with Leu, which was not observed with Y195W, in the CGTase from *B. circulans strain 251*. We also substituted Y183 of CGTase G825‐6 with Leu, which resulted in a very low synthesis of CD7 but increased CD8 and LR‐CD product shares. However, the variant Y183L showed a reduced cyclization activity and less LR‐CD were formed compared to D358R and Y183R.

To our knowledge, the variant Y183R represents the first CGTase reported with a completely suppressed synthesis of CD6 and CD7. Even at high enzyme concentrations, the variant Y183R synthesized almost solely CD8 and smaller amounts of CD9–CD12 (Figure 2). The absence of CD6 and CD7 in the reaction mixture facilitates the preparation of single LR‐CD requiring a much reduced downstream processing.

### 4.4 The CD product size spectrum of the CGTase G‐825‐6 is changed by the coupling reaction

While CGTases catalyze rather nonspecifically, the formation of cyclic products in a broad size range, the final product size spectrum of the synthesis reaction is strongly influenced by the coupling reaction of the CGTase. LR‐CD synthesized initially are thereby further transformed to linear products and smaller CD by the enzyme, while the smaller CD as more unsuitable substrates for the coupling reaction accumulate during longer reaction times (Terada et al., 2001). We could show that the variant Y183R accumulated CD8 as the smallest and most stable cyclic product while the CGTase G‐825*‐*6 accumulated higher amounts of CD7 by the coupling reaction when LR‐CD were used as substrate (Figure 3).

## CONFLICT OF INTEREST

The authors declare no conflict of interest.

## AUTHORS CONTRIBUTION

CS, SM, WZ conceived the project; CS, SM planned the experiments; CS, SM performed the experiments and analyzed data; CS, WZ wrote the paper.

## ETHICS STATEMENT

None required.

## Data Availability

Protein sequences and structures are accessible at the NCBI server (https://www.ncbi.nlm.nih.gov) or at the RCSB Protein Data Bank (https://www.rcsb.org). Accession numbers are mentioned in the text.
