# Supplementary material for: Engineered cyclodextrin glucanotransferases from Bacillus sp. G‐825‐6 produce large‐ring cyclodextrins with high specificity
Source: Microbiologyopen. 2018 Oct 25;8(6):e00757. doi: 10.1002/mbo3.757 (PMC6562119; doi:10.1002/mbo3.757)
Supplement: Supplementary file 1 [file MBO3-8-e00757-s001.pdf]

Supplementary material

Engineered cyclodextrin glucanotransferases from *Bacillus* sp. G-825-6 produce large-ring cyclodextrins with high specificity

Christian Sonnendecker, Susanne Melzer, Wolfgang Zimmermann

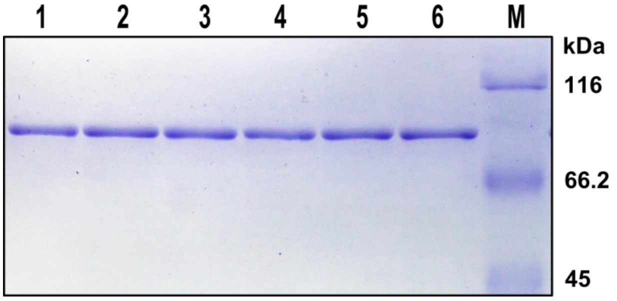

S1: SDS PAGE of purified preparations of CGTase G-825-6 and the constructed variants. Lane 1: CGTase G-825-6; Lane 2: Y183W; Lane 3: Y183R; Lane 4: D358R; Lane 5: Y183W/D358R; Lane 6: Y183W/D358R, Lane M: molecular weight marker.
